# Empirical Examinations of Effects of Three-Level Green Exercise on Engagement with Nature and Physical Activity

**DOI:** 10.3390/ijerph15020375

**Published:** 2018-02-22

**Authors:** Ke-Tsung Han, Po-Ching Wang

**Affiliations:** 1 Department of Landscape Architecture, National Chin-Yi University of Technology, 57 Sec. 2, Zhongshan Rd., Taichung City 41170, Taiwan; kthan@ncut.edu.tw; 2 Department of Landscape Architecture, National Chiayi University, 300 Syuefu Rd., Chiayi City 60004, Taiwan

**Keywords:** bodily movement speed, arm activity, limb activity, energy expenditure, environmental naturalness

## Abstract

Green exercise can be classified into three levels based on engagement with nature. Although this classification was proposed more than a decade ago, few studies have investigated it since. The present study examined the effects of green exercise levels on engagement with nature and physical activity (PA) through a field experiment. A questionnaire was distributed to 95 students from a technology university in Central Taiwan to measure their level of engagement with nature in people-environment transactions, while their PA was measured using three instruments. In addition, because social interaction may distract individual attention from activities or their environments, the present study incorporated the presence of partners as a control variable. The results revealed that (1) engagement with nature and PA significantly differed between the levels of green exercise, and the higher the level of green exercise participated in, the greater the level of engagement with nature; and (2) although the presence of partners did not influence the level of engagement with nature, it significantly affected the level of PA.

## 1. Introduction

Physical inactivity has become the fourth largest cause of death worldwide. According to the World Health Organization [1], approximately 6% of annual mortality, or more than two million deaths per year, are attributable to a sedentary lifestyle. Moreover, people in modern society face increasingly serious challenges regarding physical and psychological health and well-being [2]. Hence, physical inactivity and psychological illnesses such as depression have become public health problems [1]. Since physical activity (PA) and contact with nature are conducive to both physical and psychological health and well-being [3,4], the importance and value of conducting PA or exercise in natural environments, i.e., green exercise, should not be simply ignored.

The concept of green exercise was not proposed in academia until 2005, and few studies have investigated it since. Green exercise might be better conceptualized as PA or exercise performed in environments with a greater ratio of natural to artificial elements than that typically encountered in everyday life [5]. Therefore, green exercise is composed of both PA or exercise and the natural environment, which are both physically observable phenomena. Physical activity and exercise both involve bodily movements produced by skeletal muscles resulting in energy expenditure [6]. Therefore, green exercise encompasses all types of PA undertaken in open spaces (e.g., parks, squares, avenues, residential gardens, ranches, playgrounds, sports fields, forests, meadows, and farmland). It is not necessarily limited to organized or purposely planned sports undertaken in an area of wilderness or a nature reserve.

Since there are many types of green exercise, scholars have further distinguished it into several major categories. Some research, for instance, classifies green exercise into three levels based on the degrees of engagement with nature [4,7]. The concept of engagement with nature can be regarded as a transaction between the people, who perform the PA, and environment, where the PA takes place. The first level green exercise is viewing nature, including looking through a window and viewing surrogates of nature, such as photographs, posters, or video clips. The second level is being in nature, which may include incidental activities such as walking, cycling, and talking to someone in a park. The third and highest level is active involvement with nature through activities such as gardening, farming, and camping.

However, the concept of stratifying green exercise based on degrees of interaction with nature has long lacked support from research, and the classification of green exercise based on degrees of engagement with nature has never been empirically examined. This deficiency is perhaps due to the nature of the people-environment transaction, which is not a physically observable phenomenon. Existing empirical research focused on the tangible aspects of green exercise has revealed that exercise undertaken in natural environments provides more physical and psychological health benefits than does that undertaken in built environments. Thus, in addition to investigating the effects of various types of exercise conducted in different environments specifically on health benefits [8,9,10,11,12], researchers should also examine the effects of various types of PA, particularly the three levels of green exercise, performed in the same natural environment, on people from a more general perspective.

Moreover, social interaction during green exercise may affect participants. For example, exercising with a partner may decrease an individual’s sense of isolation or encourage interaction with natural environments [13,14]. However, social interaction or company may also divert participants’ attention from the PA being performed [15] or the environment [16]. In view of the dearth of empirical research on the aforementioned assertions, the objective of the present study was to examine the effects of the aforementioned three levels of green exercise on participants’ engagement with nature and PA in the same natural environment while incorporating social interaction as a control variable in the experiment. The main contributions of the present study in terms of the existing research gap are the clarification of whether green exercise can be stratified into different levels by their engagement with nature and a further exploration of whether social interaction, such as the presence of partners, affects three-level green exercise. The present study shows the partial results of a more complicated research project on the effects of green exercise on humans.

## 2. A Proposed Framework of Green Exercise

**Figure 1:**
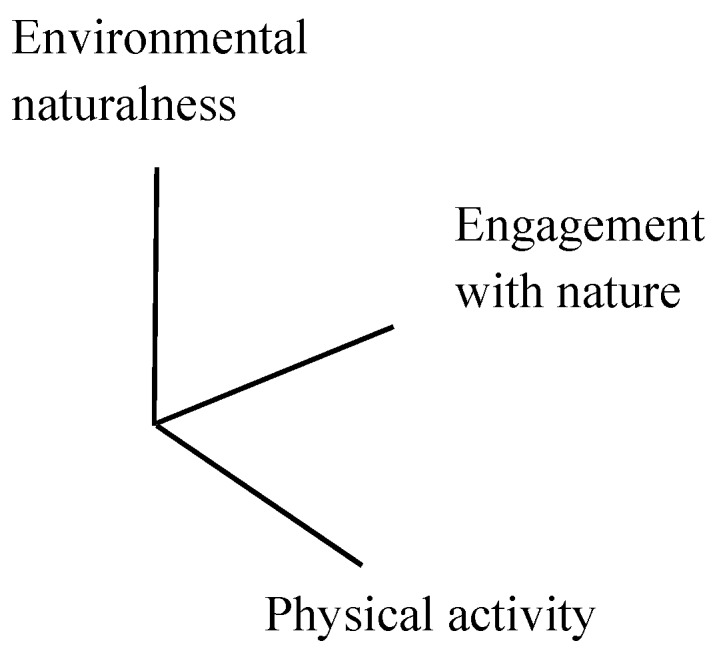
Framework of green exercise.

Based on the concept of green exercise, PA performed in natural environments, a framework composed of three basic dimensions is proposed (Figure 1). One dimension is environmental naturalness, which ranges from entirely artificial elements (zero naturalness) to no artificial elements (complete naturalness). Another dimension is PA, which ranges from completely static bodily activity (zero PA) to extremely vigorous PA. These two dimensions are orthogonal, because environmental naturalness and PA are independent. That is, people can perform any intensity of PA, in any type of environment. The other dimension is engagement with nature, which ranges from complete disengagement (zero interaction) to strong engagement (active interaction). The dimension of engagement with nature is where the natural environment and PA interact with each other. These three dimensions should all be conceptualized as a continuum rather than classified or dichotomized (e.g., artificial vs. natural, static vs. vigorous, and disengagement vs. engagement).

The interplay of the aforementioned three dimensions affects green exercise. Environmental naturalness and PA can be easily observed and objectively measured. Existing research has supported a positive effect of environmental naturalness (usually in a built-natural dichotomization) on human beings performing PA (e.g., sitting, walking, or running) [10,12,17]. However, the degree of engagement with nature should also influence the effects of undertaking PA in natural environments, since the degree of engagement with nature is a transaction between people and surrounding environments. Although it is not easily observed and measured, engagement with nature should, theoretically, play a critical role in green exercise.

The importance of engagement with nature as a people-environment transaction can be elaborated as follows. When people interact with their surroundings, exercising or not, they can take in environmental information through their senses by paying attention to the external world, or they can refuse or even ignore the external information by paying attention internally to themselves [18]. Thus, when people deliberately lower their sensing of the natural environment, rather than taking in nature by changing their attention focus, the positive effect of the environmental naturalness on physical and psychological health may be diminished. Conversely, when people deliberately lower their sensing of the built environment rather than taking it in, the negative effect of the built environments on physical and psychological health may be compromised.

Additionally, because the three-level green exercise is a subcategory of green exercise, three-level green exercise also possesses the characteristics of the three dimensions. Although the major difference between the three levels of green exercise is their degree of engagement with nature [4,7], they may also differ in PA and environmental naturalness. Nevertheless, given that engagement with nature is only one element of green exercise, despite the potential importance of green exercise, engagement with nature and green exercise are not the same. Thus, people participating in larger amounts of green exercise are not necessary experiencing more engagement with nature, since that may vary by their choice of attention focus, the environmental features, and the PA characteristics. If the environments are beautiful and interesting, as in most cases in natural settings, it is easy to draw peoples’ attention to the external world [19]. Physical effort also affects attention focus, while high-intensity, heavy workload, and long-duration PA results in internal attention [20,21].

### 2.1. Environmental Naturalness

The difference between natural and built environments is evident. Studies on human cognition in terms of environments have confirmed that the key component of distinguishing between environments is environmental naturalness [22,23]. Although the natural-built dichotomization has been employed in many quantitative studies to examine green exercise, objectively describing environmental naturalness through a continuum from natural to man-made should be more realistic and accurate than the dichotomization [24]. The naturalness of environments in the framework of green exercise may be objectively quantified using existing methods; for example, the green cover ratio refers to that between a vertically projected area covered by plants and the corresponding base area; the visible greenness ratio refers to the quantity of plants visible to human eyes; and ecological diversity refers to variety in an ecosystem. In addition, perceived naturalness is a relatively subjective approach for measuring the naturalness of an environment [25,26].

### 2.2. Physical Activity

Bouchard and Shephard [27] defined PA as any bodily movement by skeletal muscles that results in more energy expenditure than does resting. Related research has indicated that PA encompasses four characteristics: (1) bodily movement through skeletal muscles, (2) energy expenditure, (3) continuous variation of energy expenditure from low to high, and (4) positive correlation with physical fitness [6]. Methods for measuring and assessing PA can be grouped into the following seven major categories: survey procedures (e.g., diary and recall questionnaire), mechanical and electronic monitors (e.g., heart rate monitor and pedometer), calorimetry, job classification, physiological markers, behavioral observations, and dietary measures. Each method has advantages and drawbacks; validity, reliability, practicability, and convenience must be evaluated according to participant characteristics, research methodology, budget, and time constraints [28].

For green exercise, various mechanical and electronic monitors such as the Intelligent Device for Energy Expenditure and Activity (IDEEA) and actigraph units can be employed to obtain relatively accurate results of PA. Regarding energy expenditure, respirometry may be used to measure a participant’s oxygen consumption and carbon dioxide production during exercise; energy expenditure can then be calculated through a chemical equation [29,30]. Alternatively, the double-labeled water method is a more expensive but more accurate approach to estimating energy metabolism through isotopes [31]. Another gold standard method for measuring energy expenditure is direct calorimetry conducted in a controlled chamber [32].

### 2.3. Engagement with Nature

Nature experience, which refers to an expeditionary learning method that originated from outdoor education, emphasizes the expansion of lessons to outdoor environments and direct contact with nature through sight, sound, smell, taste, and touch [33]. In addition, Berger and McLeod [34] argued that nature is a medium that affects the inner essence of humans who make contact with nature experientially to reconnect with their physical, psychological, and spiritual selves. Therefore, active engagement and interaction with nature increases a participant’s satisfaction [13]. Similarly, Kurt Hahn, a Jewish German educator, devised the first outward bound program in Wales in 1941 [35]. The program emphasized the inclusion of nature in educational content and expected students’ direct contact with nature to facilitate a direct and respectful relationship with the natural world through which students could refresh their minds, bodies, and spirits [36]. Horticultural therapy refers to gardening activities with plants and related materials that improve a person’s physical, psychological, and spiritual conditions [37], and generate therapeutic effects through sight, sound, smell, taste, and touch [38]. Therefore, the degree of engagement with nature (or interaction with nature) should encompass physical, psychological, and spiritual aspects in addition to all five sensory functions. Currently, no apparent existing methods or instruments for quantifying engagement with nature in the framework of green exercise are available.

## 3. Research Methodology

### 3.1. Research Design

This study employed a field experiment because of excellent ecological validity [39]. The participants were randomly assigned into three experimental treatments in a real environment for a randomized controlled trial. The randomized assignment of the participants facilitated probabilistic equivalence among the treatment groups and eliminated most threats to internal validity [39].

### 3.2. Research Framework and Hypotheses

**Figure 2:**
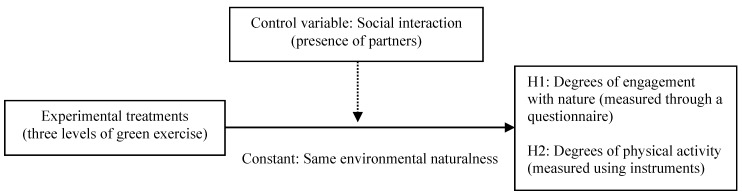
Research framework and hypotheses.

This study investigated the effects of green exercise levels on engagement with nature and PA in the same environment with the same level of environmental naturalness. That is, this study only focuses on the dimensions of engagement with nature and PA by holding the environmental naturalness dimension as a constant in order to simplify the experiment. In addition, social interaction was incorporated as a control variable. The research framework and hypotheses of this present study are displayed in Figure 2. This study proposed the following two research hypotheses: (1) Participants’ engagement with nature varies based on green exercise level, and (2) participants’ PA varies based on green exercise level. Scholars have asserted that the higher level of green exercise participated in, the greater levels of engagement with nature [4,7] and/or PA.

### 3.3. Participants and Partners

For those people participating in this experiment, their probability and severity of physical or psychological hazards were not as strong as those encountered in everyday life or routine medical, dental, and psychological examinations by healthy people in general. However, because this study sought to investigate the effects of outdoor PA on people, for safety concerns, only individuals without injuries, pain, or asthma [15], and those not allergic to sunlight, air, or plants were eligible for participation. The participants were informed that this study was to understand their experiences while conducting PA in a park.

The participants were encouraged to be accompanied by friends or family members. Although invited partners could be of either gender, they could not be a spouse or someone with a romantic relationship with the participant [15]. In addition, invited partners were not required to complete any questionnaire, wear any instrument, or provide any personal information, because the presence of partners was a control variable in the experiment. However, each participant was required to report his or her number of accompanying partners in the questionnaire. 

This study recruited 101 students from a technological university in central Taiwan. The 95 valid participants comprised 41 men and 54 women. The demographic data of these participants are listed as follows: mean age = 20.47 years (standard deviation [*SD*] = 1.236), mean height = 165.53 cm (*SD* = 8.805), mean weight = 56.96 kg (*SD* = 11.391), and mean body mass index = 20.67 (*SD* = 2.963). In addition, 39 participants were accompanied by partners, whereas 59 participated independently.

### 3.4. Experimental Settings

**Figure 3:**
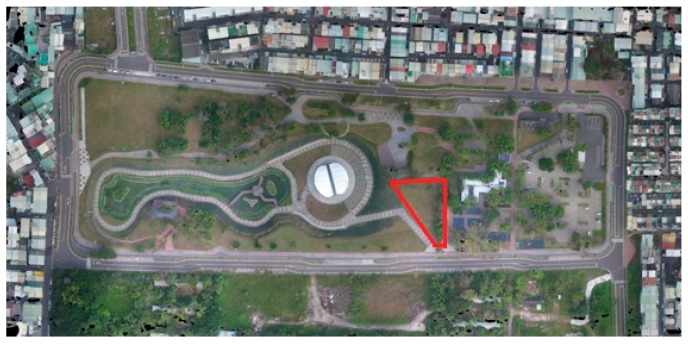
Aerial view of experiment site (the area within the solid red line).

**Figure 4:**
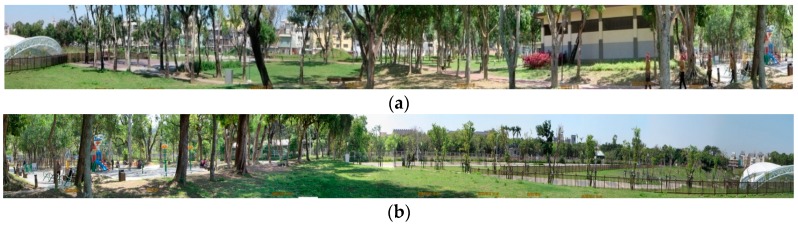
Eye-level images of experimental environment. (**a**) 180° inside-out panorama facing north; and (**b**) 180° inside-out panorama facing south.

The experiment was conducted in Pinglin Forest Park in Taiping District, Taichung City, Taiwan. The green space of 3.7 ha accounts for ≥30% of the total area of the park (11.4 ha). A small area in the park was selected to conduct the experiment. The selected area was a trapezoid-like shape with bases of approximately 15 and 80 m and legs of approximately 56 and 67 m, totaling approximately 0.24 ha, as shown in Figure 3 and Figure 4. The experiment was conducted over a near 3-month period between 2 December 2015 and 23 February 2016. The mean temperature during this period was 20.50 °C (*SD* = 3.405), with maximum and minimum temperatures of 27.97 and 12.42 °C, respectively. To accommodate the participants’ school hours, the experiment was conducted from Monday to Friday between 8:00 and 17:00. The 15-min duration of each treatment was predetermined because previous studies have revealed that a 15-min session of green exercise is sufficient for provoking physical and psychological responses [24,40].

### 3.5. Experimental Treatments

The experimental treatments were intended to operationalize the three levels of engagement with nature [4,7], which were the questions of interest along with their PA. In the experiment site, the participants were randomly assigned to perform only one of the three green exercise levels and were asked to participate in one session (i.e., no repeated participation). The 33 participants of Level 1 green exercise (13 with partners) were asked to sit on chairs in the experiment site and view the surrounding environment but were not allowed to perform any other activities. The 31 participants of Level 2 green exercise (13 with partners) were permitted to perform any activities (e.g., walking, jogging, viewing scenery) except sitting and lying down to rest. The 31 participants of Level 3 green exercise (13 with partners) were required to collect at least seven natural elements (e.g., gravel, flowers, fruit, insects) perceived as special by the participants [41]. 

### 3.6. Measuring Instruments

#### 3.6.1. Engagement with Nature

The degree of engagement with nature can be viewed as people-environment transactions. Therefore, this study measured the participants’ degree of engagement with nature using a questionnaire, which included three aspects (sensory and perceptional, psychological, and spiritual) and comprised eight specific items of “sight, sound, smell, taste, touch, body and limbs, cognition, and spirituality.” These items were measured with a question regarding how the participant described the degree of interaction with the nature on a seven-point Likert-type scale, where a high score indicated a strong engagement with nature. The internal consistency of these eight items had a Cronbach’s α of 0.765.

#### 3.6.2. Physical Activity

This study objectively evaluated the participants’ PA through the following three mechanical and electronic devices: (1) a wearable device with global positioning system (GPS) functions for measuring the speed of bodily movement, (2) an actigraph used specifically to measure arm activity, and (3) the IDEEA used to measure limb activity and energy expenditure. These three devices enabled comprehensive measurements of the participants’ PA to be taken.

**Figure 5:**
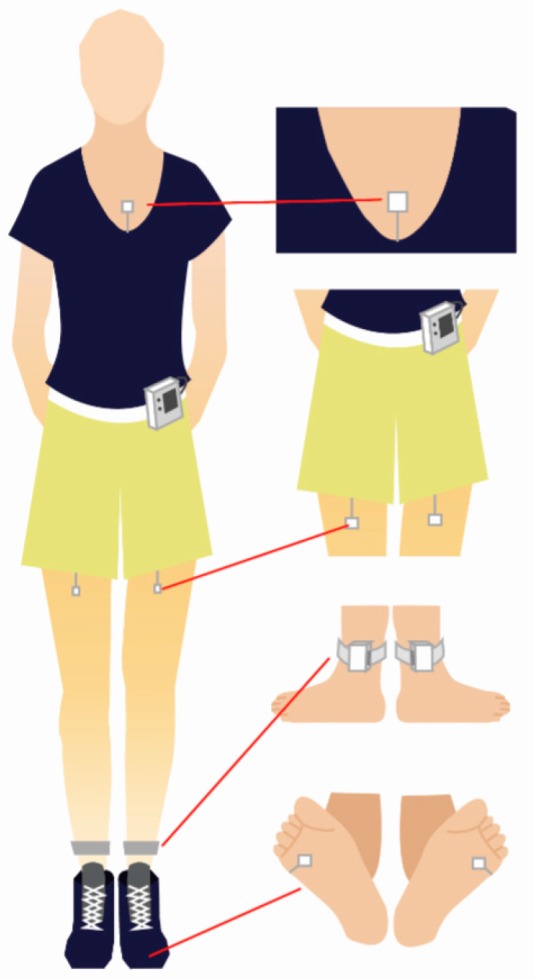
Intelligent Device for Energy Expenditure and Activity (IDEEA) 3 (cf. MiniSun LLC).

1. **Bodily movement speed:** This study asked each participant to wear a Garmin Forerunner 405 (New Taipei City, Taiwan), a GPS-enabled watch with a positioning accuracy of <10 m, on the nondominant hand to continuously monitor his or her location, path, and time. After the data were uploaded to ArcGIS, a geographic information system, each participant’s bodily movement speed could be calculated by dividing the total distance traveled by time.
2. **Arm activity:** The actigraph employed in this study was the MicroMini Motionlogger Actigraph (Ambulatory Monitoring Inc., New York City, NY, USA), the appearance of which resembles a wristwatch. The device was worn on each participant’s dominant hand to measure his or her arm activity in a high proportional integral mode [42]. Generally, actigraphy features excellent validity and reliability [43,44,45,46,47].
3. **Limb activity and energy expenditure:** This study employed the IDEEA 3 (MiniSun LLC, Fresno, CA, USA) to measure the participants’ limb activity and energy expenditure. This motion-sensing device features five biaxial accelerometers (two are wireless), which were attached to each participant’s upper torso, both thighs, and both soles with surgical Micropore tape. The data collected from these sensors were sent to a recorder worn on the waist, as shown in Figure 5. Empirical studies using the IDEEA have revealed that (1) the device can detect PA to an accuracy of ≥98% and a correlation coefficient of 0.986 between predicted and actual walking and running speeds [48]; (2) the intraclass correlation between gait and force plate measurements ranged between 0.998 (cadence) and 0.784 (step length) [49]; and (3) the IDEEA can effectively estimate energy expenditure to an accuracy of ≥95% [50].

## 4. Results

### 4.1. Engagement with Nature with Respect to Level of Green Exercise

A two-factor analysis of variance (ANOVA), where the dependent variable was the total score of engagement with nature obtained from the questionnaire and the independent variables were the experimental treatment (i.e., three-level green exercise) and social interaction (i.e., presence of partners), was conducted to test Hypothesis 1. The results revealed that (1) the interaction between experimental treatment and social interaction on engagement with nature did not reach significance (*F*_(2,89)_ = 1.243, *p* = 0.293, η*_p_*^2^ = 0.027); (2) the main effect of social interaction on engagement with nature was not significant (*F*_(1,89)_ *=* 3.328, *p* = 0.071, η*_p_*^2^ = 0.036); and (3) the main effect of experimental treatment on engagement with nature reached significance (*F*_(2,89)_ *=* 3.728, *p* = 0.028, η*_p_*^2^ = 0.077), with a moderate effect size, and zero was not included within the 95% confidence interval for the difference. Table 1 shows that the participants of Level 3 green exercise (*M* = 34.42) significantly outscored those of Level 2 green exercise (*M* = 28.97) in terms of engagement with nature (*p* = 0.010).

### 4.2. Physical Activity with Respect to Level of Green Exercise

To test Hypothesis 2, this study conducted two-factor ANOVAs, where the dependent variables were PA indicators comprising bodily movement speed measured by the GPS-enabled watch, arm activity measured by the actigraph, and limb activity and energy expenditure measured by the IDEEA, whereas the independent variables were experimental treatment (i.e., three-level green exercise) and social interaction (i.e., presence of partners). The statistical analysis results are described as follows.

#### 4.2.1. Movement Speed

Since bodily movement speed violated the assumption of homogeneity and the numerical values of the raw data all exceeded zero, a log transformation on the raw data was performed. The results revealed that (1) the interaction between experimental treatment and social interaction on movement speed did not reach significance (*F*_(2,89)_ = 1.810, *p* = 0.170*,* η*_p_*^2^ = 0.039); (2) the main effect of social interaction on movement speed was not significant (*F*_(1,89)_ = 0.097, *p* = 0.757, η*_p_*^2^ = 0.001); and (3) the main effect of experimental treatment on movement speed reached significance (*F*_(2,89)_ = 94.590, *p* = 0.000, η*_p_*^2^ = 0.680), with a large effect size, and zero was not included within the 95% confidence interval for difference. Table 2 shows that the participants of Level 3 green exercise (*M*_log_ = −4.639) significantly outpaced those of Level 1 green exercise (*M*_log_ = −1.254) in terms of movement speed (*p* = 0.000). Additionally, the participants of Level 2 green exercise significantly outpaced those of Level 1 green exercise (*M*_log_ = −4.598) (*p* = 0.000).

#### 4.2.2. Arm Activity

Since the data collected from the actigraph violated the assumption of homogeneity even after every possible transformation, this study had to resort to the nonparametric Kurskal-Wallis one-factor ANOVA ranks (also known as the *H* test). All data collected from the actigraph were converted from numerical values to ranks according to their order. Subsequently, the ranks in each group were aggregated to measure the difference between mean and expected values among the treatment groups [51]. The mean ranks for Treatments 1 to 3 were 20.697, 46.290, and 48.451, respectively (chi-square = 51.666, degrees of freedom = 1). The two-tailed test results demonstrated an asymptotic significance between the actigraph data among the treatment groups (*p* = 0.000), as shown in Table 3. The results of the post-hoc comparisons indicated that, in terms of arm activity, the participants of Level 2 and 3 green exercise significantly outperformed the Level 1 participants, and that no significant difference was observed between the Level 2 and 3 participants (Table 3).

#### 4.2.3. Limb Activity and Energy Expenditure

Because the participants performing Level 1 green exercise were asked only to view nature from a sitting position, their IDEEA data all approximately zero. Consequently, a comparison was only possible between the Level 2 and 3 green exercise participants. Since the data collected by IDEEA contained multiple variables of limb activity, the family-wise error rate (i.e., α divided by the number of dependent variables) was employed in the multivariate ANOVAs (MANOVAs). The results revealed that (1) the main effect of experimental treatment on upstairs or downstairs momentum reached significance (*F*_(1,58)_ = 9.538, *p* = 0.003, η*_p_*^2^ = 0.141), with a high effect size, and zero was not included within the 95% confidence interval for the difference, while the Level 2 green exercise participants (*M* = 84.300) were significantly greater than those of Level 3 green exercise (*M* = 66.525) (*p* = 0.003); (2) the main effect of experimental treatment on upstairs or downstairs calorimetry reached significance (*F*_(1,58)_ = 6.977, *p* = 0.011*,* η*_p_*^2^ = 0.107), with a moderate effect size, and zero was not included within the 95% confidence interval for the difference, while the Level 2 green exercise participants (*M* = 4.378) were significantly greater than those of Level 3 green exercise (*M* = 3.506) (*p* = 0.011); (3) the main effect of experimental treatment on the number of posture transitions nearly reached significance (*F*_(2,89)_ = 3.99, *p* = 0.05*,* η*_p_*^2^ = 0.064), with a moderate effect size, and zero was included within the 95% confidence interval for the difference, while the Level 3 green exercise participants (*M* = 12.030) were almost significantly greater than those of Level 2 green exercise (*M* = 4.650) (*p* = 0.050); (4) the interaction between experimental treatment and social interaction on the number of sitting posture reached significance (*F*_(2,89)_ = 4.246, *p* = 0.044*,* η*_p_*^2^ = 0.068), with a moderate effect size, and zero was not included within the 95% confidence interval for the difference. In addition, the follow-up tests demonstrated that the number of sitting posture for Level 3 green exercise participants without partners (*M* = 27.167) was significantly greater than that of Level 2 green exercise participants without partners (*M* = 8.611) (*p* = 0.001; Table 4); and (5) no other significant differences were observed.

## 5. Discussion

### 5.1. Green Exercise Level and Engagement with Nature

Hypothesis 1 was not rejected. The Level 3 green exercise participants were more significantly engaged with nature than were those of Level 2 green exercise. In contrast to free activities without a specific purpose, collecting natural elements in the same environment favored the sensory, cognitive, and psychological and spiritual aspects of engagement with nature. However, the Level 1 green exercise participants’ engagement with nature was not significantly lower than those of the Level 2 and 3 participants. This may be attributed to the fact that the Level 1 participants were in a sitting position without participating in any activity, so they were able to concentrate on viewing the environment without distraction from other activities [16,21]. Since sight is the most crucial sensory function for humans [52], the Level 1 participants’ engagement with nature may not have been lower than those of the Level 2 and 3 participants.

The presence of partners had no effect on engagement with nature. Although previous studies have asserted that social interaction may distract the participants’ attention from the activities [15] or the environment [16], engagement with nature, distraction, and attention focus may be different, but interrelated concepts. Another concept similar to engagement with nature is *fascination* from the attention restoration theory (ART) proposed by Kaplan and Kaplan [19]. Fascination generally refers to attention and interest drawn to natural objects, content, events, or processes [53,54] that stimulate joyful feelings through aesthetics [55]. Fascination is also one type of people-environment transactions [19]. Future studies could further examine the interrelationships between these concepts.

Additionally, with regard to the ART, Kaplan and Kaplan [19] held the opinion that, from the point of view of human evolution, an individual’s interactions with natural environments, particularly those that are compatible with their goals or purposive activities, are large enough to be explored and experienced physically and/or conceptually. They also provide opportunities for escape from attention demands, which can help reduce the individual’s cognitive load and facilitate psychological and mental recovery, and therefore can improve their well-being. In this study, participants who engaged in level three exercises were found to have a higher degree of engagement with nature. Thus, the promotion of higher levels of green exercise with appropriate physical effort [20,21] may help to optimize the psychological health of participants.

### 5.2. Green Exercise Level and Physical Activity

Hypothesis 2 was not rejected. Some PA degrees of higher level green exercise were significantly greater than those of lower level of green exercise. First, the participants performing Level 2 and 3 green exercises exhibited significantly faster bodily movement speeds than did the Level 1 participants. However, because no significant difference in bodily movement speed between Level 2 and 3 participants was observed, the difference in PA between free activities without a specific purpose and collecting natural elements was indistinguishable. This may be attributed to the relatively small area where the experiment was conducted, which was unsuitable for fast movement activities such as running. Second, although the Level 2 and 3 participants significantly outperformed the Level 1 participants in terms of arm activity, no significant difference was observed between the Level 2 and 3 participants. Therefore, the difference in arm activity between free activities without a specific purpose and collecting natural elements was also indistinguishable. Third, the Level 2 participants significantly outscored the Level 3 participants in upstairs or downstairs momentum and calorimetry, indicating that the Level 2 participants with fewer movement constraints raised their feet more often and burned more calories than did the Level 3 participants. By contrast, the Level 3 participants outscored the Level 2 counterparts in terms of number of posture transitions, indicating more posture changes were required to collect natural elements than for free activities without a specific purpose. Furthermore, the Level 3 participants without partners had a significantly greater number of sitting postures than that of the Level 2 participants. This may be attributed to sitting or crouching positions possibly favoring the comfortable collection of natural elements, whereas the Level 2 participants were instructed not to sit or lie down. However, the participants with partners present tended to decrease their sitting or crouching frequency to maintain interactions with their partners, resulting in no significant difference being observed in the number of sitting posture between Level 2 and 3 participants with partners.

### 5.3. Limitations and Suggestions

Due to the lack of similar research, this study featured an explorative characteristic, and replications are encouraged in future studies. The three levels of green exercise (viewing nature in a sitting position, free activities except for sitting and lying down, and collecting seven natural elements) selected in this study may not be the most representative activities and/or the most appropriate operationalization of the engagement with nature. In addition, although empirical results have confirmed the reliability (Cronbach’s α = 0.765 only) and the sensitivity of the questionnaire items proposed in this study in terms of engagement with nature, further examinations regarding validity and reliability are required before a formal scale is developed [56,57,58]. Subsequent studies could, then, seek to verify whether the people-environment transactions of engagement with nature are potential mediating variables between green exercise and its influence on physical and psychological responses.

Because of the complexity of human bodily movements, this study employed three devices to perform real-time and objective measurements on bodily movement speed, arm activity, and limb activity and energy expenditure. Nevertheless, the use of separate equipment during the field experiment created inconvenience and increased the likelihood of errors in operating procedures. Future studies should use integrated instruments to quantify PA. For the measurements of engagement with nature, in addition to questionnaire surveys, magnetoencephalography (MEG), functional magnetic resonance imaging (fMRI), and magnetic resonance spectroscopy (MRS) can be used to explore in-depth brain activity in response to people-environment transactions. However, these instruments are difficult for real-time and in situ measurements of PA.

Furthermore, because all participants in this study were students of a technology university, the research findings may not be generalizable to other occupations, age, and ethnicity groups. Additionally, the experiment was conducted in a confined area of 0.24 ha, which might have limited the participants’ PA and prevented diverse and in-depth interaction with the natural environment. Subsequent studies could consider conducting experiments in more spacious areas. Finally, the environmental naturalness was held constant in this experiment. Further research may consider the conducting of tests in which varying degrees of environmental naturalness are examined. Hence, the interplays of the three dimensions of the proposed framework could be integrated to allow for a more comprehensive and thorough understanding of green exercise.

## 6. Conclusions

This study pioneered the investigation of the effect of three-level green exercise on engagement with nature and PA through a field experiment. Because of the lack of literature for reference, this study featured an explorative characteristic. Nevertheless, the results of this study supported the three-level green exercise hierarchy. The three green exercise levels can indeed be classified with respect to engagement with nature [4,7] and PA, a preliminary demonstration of the usefulness of the proposed green exercise framework. In addition, because this study specifically incorporated social interaction (i.e., the presence of partners) as a control variable, the research results verified the importance of this variable. Although the presence of partners did not affect the participants’ engagement with nature, it did affect the participants’ PA in terms of sitting and crouching. Given that green exercise can provide the synergistic health benefits of contact with nature and PA [59], its potential for public health deserves more attention.

## Figures and Tables

**Table 1:** Summary of two-factor ANOVA of engagement with nature ^a^.

Effect	*F*	*p*	η*_p_*^2^	Treatment (*n*/*N*) ^b^	Mean (*SD*)	Mean Diff.	95% Conf. Int. for Diff.		Post-hoc
							Lower Bound	Upper Bound	
Treatment ^c^ × Social interaction ^d^	1.243	0.293	0.027	I (33/95)	31.61 (6.45)	I−II I−III II−III	−1.72 −7.17 −9.88	7.00 1.55 −1.02	III > II (*p* = 0.010)
				II (31/95)	28.97 (8.61)				
Treatment (I, II, III)	3.728	0.028 *	0.077						
				III (31/95)	34.42 (6.53)				
Social interaction (Presence of partners)	3.328	0.071	0.036	Yes (39/95)	33.26 (6.68)				
				No (56)	30.55 (7.90)				

^a^ The interaction between experimental treatment and social interaction is accounted for. ^b^ *N* denotes the total number of participants (95 excluding partners), whereas *n* denotes the number of those in the subpopulation (excluding partners). ^c^ I denotes Level 1 green exercise; II denotes Level 2 green exercise, and III denotes Level 3 green exercise. ^d^ Social interaction denotes the presence of partners. * denotes significance level at 0.05.

**Table 2:** Summary of two-factor ANOVA of movement speed (log transformation) ^a^.

Effect	*F*	*p*	η*_p_*^2^	Treatment (*n*/*N*) ^b^	Mean (*SD*)	Mean Diff.	95% Conf. Int. for Diff.		Post-hoc
							Lower Bound	Upper Bound	
Treatment ^c^ × Social interaction ^d^	1.810	0.170	0.039	I (33/95)	−1.254 (0.337)	I−II I−III II−III	−0.950 −0.946 −0.154	−0.639 −0.635 0.162	I < II, III (*p* = 0.000)
				II (31/95)	−4.598 (0.246)				
Treatment (I, II, III)	94.590	0.000 ***	0.680						
				III (31/95)	−4.639 (0.141)				
Social interaction (Presence of partners)	0.097	0.757	0.001	Yes (39/95)	−0.715 (0.368)				
				No (56)	−0.753 (0.511)				

^a^ The interaction between experimental treatment and social interaction is accounted for. ^b^ *N* denotes the total number of participants (95 excluding partners), whereas *n* denotes the number of those in the subpopulation (excluding partners). ^c^ I denotes Level 1 green exercise; II denotes Level 2 green exercise, and III denotes Level 3 green exercise. ^d^ Social interaction denotes the presence of partners. *** denotes significance level at 0.001.

**Table 3:** Summary of one-factor ANOVA ranks on arm activity.

Treatment ^a^	ΣR ^b^	$\bar{R}$	$X^{2}$	*p*	Post-hoc	Sig.
I	683	20.697	51.666	0.000	$\left|{\bar{R}}_{1}-{\bar{R}}_{2}\right|=\left|25.593\right|>16.508\;\left(\mathrm{Critical}\right)$	N
II	1435	46.290			$\left|{\bar{R}}_{1}-{\bar{R}}_{3}\right|=\left|27.755\right|>16.508\;\left(\mathrm{Critical}\right)$	N
III	1502	48.451			$\left|{\bar{R}}_{2}-{\bar{R}}_{3}\right|=\left|2.161\right|<16.764\;\left(\mathrm{Critical}\right)$	Y

^a^ I denotes Level 1 green exercise; II denotes Level 2 green exercise, and III denotes Level 3 green exercise. ^b^ All raw data were measured using an actigraph; ΣR = aggregated ranks, $\bar{R}$ = mean ranks.

**Table 4:** Summary of two-factor MANOVAs on limb activity and energy expenditure ^a^.

Variable	Effect	*F*	*P*	η*_p_*^2^	Treatment (*n*/*N*)	Mean (*SD*)	Mean Diff.	95% Conf. Int. for Diff.		Post-hoc
								Lower Bound	Upper Bound	
Upstair/downstair momentum	Treatment ^b^ × Social interaction ^c^	0.314	0.578	0.005	II (31/95)	84.300 (22.764)	II−III	6.442	30.177	III < II (*p* = 0.003)
	Treatment (I, II, III)	9.538	0.003 **	0.141	III (31/95)	66.526 (23.222)				
	Social interaction (Presence of partner)	1.470	0.230	0.025	Yes (26/62)	71.238 (25.625)				
					No (36/62)	78.428 (23.558)				
Upstair/downstair calorimetry	Treatment ^b^ × Social interaction ^c^	0.077	0.783	0.001	II (31/95)	4.378 (1.383)	II−III	0.215	1.558	III < II (*p* = 0.011)
	Treatment (I, II, III)	6.977	0.011 *	0.107	III (31/95)	3.506 (1.223)				
	Social interaction (Presence of partner)	2.114	0.151	0.035	Yes (26/62)	3.659 (1.377)				
					No (36/62)	4.147 (1.343)				
Number of posture transitions	Treatment ^b^ × Social interaction ^c^	3.708	0.059	0.060	II (31/95)	4.650 (5.364)	II−III	−12.799	0.013	II < III (*p* = 0.050)
	Treatment (I, II, III)	3.99	0.050	0.064	III (31/95)	12.030 (17.209)				
	Social interaction (Presence of partner)	1.334	0.253	0.022	Yes (26/62)	6.190 (7.250)				
					No (36/62)	9.890 (16.101)				
Number of sitting posture	Treatment ^b^ × Social interaction ^c^	4.246	0.044 *	0.068	II (31/95)	10.260 (11.081)	II−II	−18.324	−2.539	II < III (*p* = 0.010)
	Treatment (I, II, III)	7.00	0.010 **	0.108	III (31/95)	22.000 (19.313)				
	Social interaction (Presence of partner)	1.133	0.292	0.019	Yes (26/62)	13.690 (14.507)				
	Interaction (No partner)	12.565	0.001 ***	0.270	No (36/62)	17.890 (18.114)				
					II (No partner) (18/36)	8.611 (11.126)	II−III	−29.194	−7.918	II < III (*p* = 0.001)
					III (No partner) (18/36)	27.167 (19.221)				

^a^ The interaction between experimental treatment and social interaction is accounted for. ^b^ *N* denotes the total number of participants (95 excluding partners), whereas *n* denotes the number of those in the subpopulation (excluding partners). ^c^ I denotes Level 1 green exercise; II denotes Level 2 green exercise, and III denotes Level 3 green exercise. *, **, *** denote significance levels at .05, 0.01, and 0.001, respectively.
